# Development and Clinical Validation of an Entrustable Professional Activities Framework for Respiratory Nurses: A Longitudinal Workplace-Based Study

**DOI:** 10.3390/healthcare14152426

**Published:** 2026-08-06

**Authors:** Yufei He, Kouying Liu, Jiaxuan Li, Hao Huang, Yan Ji

**Affiliations:** 1 School of Nursing, Nanjing Medical University, Nanjing 211166, China; heyflht@sina.com (Y.H.); ljxjx01@163.com (J.L.); 2 Department of Respiratory and Critical Care Medicine, The First Affiliated Hospital of Nanjing Medical University, Nanjing 210029, China; kouyingliu@njmu.edu.cn; 3 Department of Nursing, Wuxi People’s Hospital Affiliated to Nanjing Medical University, Wuxi 214023, China; haohuang@njmu.edu.cn

**Keywords:** respiratory therapy, clinical competence, entrustable professional activities, workplace-based assessment, generalizability theory, learning curve, longitudinal studies

## Abstract

**Highlights:**

**What are the main findings?**

- A 13-item EPA framework for respiratory nurses was developed through a two-round Delphi process, with strong expert consensus.
- Learning speeds varied across EPAs, with EPA11 showing the fastest improvement and EPA2 the slowest; the observed WBA protocol achieved acceptable reliability.

**What are the implications of the main findings?**

- The framework provides a quantifiable tool for competency assessment and progression monitoring in respiratory nursing training.
- The differential learning trajectories provide empirical evidence for designing stratified training programs.

**Abstract:**

**Background:** Entrustable professional activities (EPAs) are increasingly used in competency-based assessment, but evidence remains limited for nursing workplace-based assessment (WBA) systems that can capture learning trajectories while providing defensible reliability for programmatic decisions. **Objective:** The objective of this study is to develop a respiratory nursing EPA framework and clinically validate core EPAs using longitudinal WBA data, including performance progression, attainment patterns, and reliability requirements based on generalizability (G) theory and decision (D) studies. **Methods:** This study included two phases: (1) EPA framework development using evidence synthesis and a two-round Delphi consultation and (2) longitudinal clinical validation using WBA data. In the validation phase, 30 respiratory nurses were assessed by five trained raters over 12 weeks on four core EPAs (EPA1, EPA2, EPA5, and EPA11) using a 1–5 entrustment scale (attainment defined as a score ≥ 4). Learning trajectories were modeled using a negative exponential function (learning speed indexed by β0). Reliability was evaluated using a crossed-facet G-study framework (person × rater × occasion) with mixed-effects modeling, and D-studies were used to identify minimal rater–occasion configurations meeting EP^2^ thresholds. **Results:** In the Delphi phase, 21 experts completed both rounds (100% response rate), with high authority coefficients (Cr = 0.841 and 0.843) and significant inter-expert agreement (Kendall’s W = 0.504 and 0.532; both *p* < 0.01), yielding a final 13-item respiratory nursing EPA framework. In the validation phase, 1392 core EPA assessments were analyzed, and event-level attainment ranged from 72.5% to 89.1%. Learning speeds were heterogeneous (β0 = 0.038–0.156), with the fastest improvement in EPA11 and the slowest in EPA2. In the G-study, residual variance accounted for 65.05% of the total variance, followed by person (18.21%) and occasion/week (10.13%) effects, and the main effect on rater was small (0.14%). Under the observed protocol (five raters and approximately four ratings/week), reliability reached acceptable levels (G = 0.792; EP^2^ = 0.712). The minimum D-study configurations were one rater × 11 ratings/week for EP^2^ ≥ 0.70 (EP^2^ = 0.703) and two raters × 12 ratings/week for EP^2^ ≥ 0.80 (EP^2^ = 0.812). **Conclusions:** The respiratory nursing EPA framework showed strong expert-consensus support and promising longitudinal clinical validation evidence. A core EPA-based WBA approach demonstrated developmental sensitivity and acceptable dependability under feasible sampling plans, supporting its use for structured competency assessment and progression monitoring in respiratory nursing training.

## 1. Introduction

Respiratory nursing routinely involves high-acuity decision-making and technically demanding interventions, including oxygen therapy titration, airway clearance, ventilatory support management, and individualized rehabilitation planning. In such settings, competency evaluation must reflect not only what nurses know but also what they can perform safely and consistently in authentic clinical contexts. However, many nursing competency assessment approaches still rely on knowledge-based testing or fragmented procedural checklists, which may not adequately capture integrated workplace performance, progressive development, or readiness for increased clinical autonomy [1,2].

Entrustable professional activities (EPAs) were introduced to operationalize competency-based education by translating abstract competencies into observable units of professional work that can be entrusted once sufficient performance is demonstrated under supervision [3,4]. EPA-based assessment emphasizes direct observation, structured entrustment ratings, and longitudinal aggregation of multiple low-stakes assessments to support both formative feedback and programmatic decisions [5]. EPAs have been widely adopted across health professions education and increasingly explored in nursing contexts to strengthen workplace-based assessment and clarify expectations for role-specific practice [6,7].

EPAs have been adopted across health professions education and are increasingly explored in nursing and advanced practice nursing to strengthen workplace-based assessment and clarify expectations for role-specific practice [8,9]. Recent nursing education studies have reported framework development, qualitative evaluation, pilot programmatic EPA assessment in nurse practitioner training, and modified Delphi development for nursing EPAs in emerging contexts (e.g., telemedicine and long-term care) [10,11]. Nevertheless, specialty nursing EPAs remain context-dependent, and transferability across institutions and clinical systems can be limited by differences in practice scope, patient mix, workflow, and supervision structures [1,12].

Despite growing interest, several gaps remain for specialty nursing EPAs, particularly in respiratory nursing. First, EPA frameworks are often context-dependent, as practice scope, training pathways, patient mix, and service organization vary across systems and institutions, limiting direct transferability from one setting to another [2,13]. Second, much of the nursing EPA literature focuses on framework development or cross-sectional evaluations, whereas longitudinal evidence using real workplace assessment data is comparatively scarce [14,15]. Third, without longitudinal validation, it is difficult to determine whether EPA-based assessment sensitively captures competence acquisition over time, whether attainment benchmarks are meaningful, and how much sampling is needed to support dependable decisions in real clinical environments [6,16].

A central challenge in workplace-based assessment is that score variability arises from multiple sources—learner differences, rater effects, occasion/week effects, and their interactions—alongside substantial residual variance reflecting case-mix, context, and random errors. Generalizability theory offers a principled approach to decompose variance across facets and quantify reliability as a design problem rather than as a fixed test property [17,18]. By extending G-studies to decision (D) studies, educators can determine feasible combinations of rater number and observation frequency required to achieve reliability targets. In parallel, longitudinal learning-curve modeling provides an interpretable framework for quantifying progression, identifying differences in learning speed across tasks, and supporting feedback through trajectory-based insights [19]. By integrating these approaches, we can generate both psychometric evidence (dependability) and educational evidence (growth patterns) from the same workplace dataset, thus directly informing implementation and resource planning.

Therefore, the present study aimed to develop and clinically validate a core EPA-based workplace assessment framework tailored to respiratory nursing using longitudinal real-world evaluation data collected over 12 weeks. Specifically, we sought to (1) summarize performance and attainment patterns for four core EPAs (EPA1: implement oxygen therapy; EPA2: perform mechanical airway-clearance techniques; EPA5: implement non-invasive ventilation; and EPA11: develop individualized pulmonary rehabilitation prescriptions) using an attainment benchmark with a score ≥ 4; (2) characterize learning trajectories using negative exponential learning-curve modeling and compare learning speeds across EPAs; and (3) evaluate reliability using generalizability theory and decision studies to identify minimal feasible rater-by-occasion sampling designs that achieve acceptable dependability (EP^2^ ≥ 0.70 and ≥0.80). We hypothesized that core EPA performance would demonstrate measurable longitudinal improvement and that acceptable dependability could be achieved under feasible sampling plans suitable for real clinical training environments [17,19].

## 2. Materials and Methods

### 2.1. Study Design, Setting, and Ethics

This study comprised two phases: (i) development of respiratory nursing entrustable professional activities (EPAs) using evidence synthesis and Delphi-based expert consultation and (ii) longitudinal clinical validation of selected core EPAs using workplace-based assessment (WBA) data. Clinical validation was conducted from February to April 2025 in four respiratory units across two campuses of a tertiary general hospital, and the study protocol was approved by the institutional ethics committee (approval No. WXEY-2025-113, Date 30 July 2025).

### 2.2. Participants

A convenience sample of 30 N1-level respiratory nurses was enrolled for clinical validation. In the context of the Chinese nursing career ladder system, the N1 level denotes the second tier of clinical nursing competency, typically representing nurses who have completed their initial orientation and basic training. These nurses generally possess 1 to 3 years of full-time clinical experience in their specialty and are capable of independently performing routine nursing tasks under indirect supervision; however, they still require guidance for more complex clinical situations. All the participants provided written informed consent prior to enrollment and participation.

### 2.3. Raters and Training

The five raters included one provincial-level respiratory specialist nurse, one municipal nursing association respiratory committee member, two respiratory ward head nurses, and one senior clinical preceptor, with all the members having more than 10 years of clinical experience in respiratory care. They also received standardized training prior to formal evaluation. The training covered EPA principles, the operational meanings of the indicators, and scoring rules of the entrustment–supervision scale, with calibration-oriented discussions of representative scenarios to enhance rating consistency and reduce construct-irrelevant variability [20]. Given evidence that assessor cognition can contribute to score variability in performance assessments, rater training was performed to emphasize shared interpretation of entrustment levels and consistent use of narrative anchors, with the rater panel characteristics summarized in Table S1.

### 2.4. Core EPAs, Entrustment Scale, and Assessment Procedures

Based on the 13-item EPA framework developed through expert panel meetings, the present study selected four core EPAs for clinical validation. The selection process followed Violato’s principles for identifying core EPAs [21], which prioritize tasks that are ① most frequently performed in daily respiratory nursing practice; ② representative of core respiratory nursing work; and ③ clearly observable and easy to evaluate. Research team members independently scored each EPA against these criteria, and items with >70% agreement among experts were considered for inclusion [22]. This threshold aligns with common practice in Delphi-based consensus studies. As a result, four core EPAs (EPA1, EPA2, EPA5, and EPA11) were selected for clinical validation. Performance was rated using a 1–5 entrustment scale (revised Chen entrustment scale) [4]. This scale defines five levels of entrustment: Level 1 (observation only, no participation), Level 2 (completion of tasks under direct supervision), Level 3 (independent performance with indirect supervision required), Level 4 (performance under remote or on-demand supervision), and Level 5 (capable of supervising others). This 5-level structure follows the original framework proposed by Chen et al. [4]. Attainment was defined a priori as an entrustment score ≥ 4, as this threshold corresponds to the level of “performance under remote/on-demand supervision” on the Chen scale, which is widely interpreted as the minimum level required for entrustment decisions in EPA-based assessment. Operational definitions of EPA1–EPA13 are provided in Table 1.

Assessments were conducted continuously over 12 study weeks. The target frequency was approximately four assessments per nurse per week, with weekly coverage of all four core EPAs. The evaluation process strictly follows the pre-defined evaluation standards and scoring procedures, and a systematic hierarchical training plan was formulated by the nursing department, providing institutional support for the cultivation of N1-level nurses. According to this plan, the nursing department and the internal medicine department organize OSCE assessments every month to evaluate the comprehensive clinical abilities of nurses. At the same time, the Department of Respiratory and Critical Care Medicine conducts special assessments for N1-level nurses every week and every month. The specific evaluation plans are detailed in Table 2 and Table 3. Assessment modalities included direct observation, mini-CEX-style scenario assessment, and objective structured clinical examination (OSCE). After each assessment, raters provided immediate structured feedback and discussed concrete actions to support progression to the next entrustment level.

### 2.5. Data Management and Cleaning

Data were standardized using prespecified rules by harmonizing nurse ID, rater ID, study week, EPA code, and score formatting. Records were excluded if they had missing key fields, out-of-range scores (<1 or >5), invalid week indices (≤0), or were not among the four core EPAs.

Duplicate records were defined as repeated entries with the same nurse–rater–week–EPA combination. When timestamps were available, the most recent record was retained, while the mean score within the duplicate cluster was used when they were unavailable. All the cleaning steps were recorded in an auditable log to ensure traceability and reproducibility.

### 2.6. Statistical Analysis

All statistical analyses were conducted using R software (version 4.5.1; R Foundation for Statistical Computing, Vienna, Austria). A two-sided significance level of 0.05 was used where applicable. Data management and visualization were performed using the following R packages: readxl, dplyr, tidyr, ggplot2, lme4, and openxlsx (all packages are freely available via the Comprehensive R Archive Network, CRAN). Analyses comprised descriptive statistics, learning-curve modeling using a negative exponential growth model, generalizability theory (G-study and D-study), and decision studies.

#### 2.6.1. Descriptive Statistics and Attainment Metrics

Descriptive statistics were used to summarize assessment volume, assessments per nurse, weekly assessment frequency, and attainment rates. Continuous variables are presented as the mean (standard deviation, SD), while categorical variables are shown as n (%), unless otherwise specified. Attainment was defined as a score ≥ 4 on the 1–5 entrustment scale.

#### 2.6.2. Learning-Curve Modeling (Negative Exponential Model)

Based on Thurstone learning curve theory [23], weekly mean scores for each core EPA were fitted using a negative exponential growth model:

$$Score\left(t\right)=Asym-\left(Asym-Start\right)e^{-\beta_{0}t}$$

where *t* denotes the study week, *Start* is the initial level, *Asym* is the asymptotic level, and *β*_0_ represents the learning speed (larger values indicate faster improvement). We report *β*_0_ and model diagnostics, including pseudo-R^2^, AIC, BIC, and Shapiro–Wilk testing of model residuals.

#### 2.6.3. Generalizability Theory (G-Study) and Decision Study (D-Study)

Reliability was evaluated using a crossed-facet generalizability (G) study framework implemented with REML mixed-effects modeling to accommodate unbalanced WBA data. The analytic structure considered person, rater, and occasion (week) facets and their interactions. Because the four core EPAs were analyzed jointly in the primary G-study, EPA was included as a fixed effect to adjust for between-EPA mean differences in entrustment scores.

Variance components were estimated using REML for the following facets: $\sigma_{p}^{2}$, $\sigma_{r}^{2}$, $\sigma_{o}^{2}$, $\sigma_{pr}^{2}$, $\sigma_{po}^{2}$, $\sigma_{ro}^{2}$, and $\sigma_{e}^{2}$.

$$G=\frac{\sigma_{p}^{2}}{\sigma_{p}^{2}+\frac{\sigma_{pr}^{2}}{n_{r}}+\frac{\sigma_{po}^{2}}{n_{o}}+\frac{\sigma_{e}^{2}}{n_{r}n_{o}}}$$

$$EP^{2}=\frac{\sigma_{p}^{2}}{\sigma_{p}^{2}+\frac{\sigma_{r}^{2}}{n_{r}}+\frac{\sigma_{o}^{2}}{n_{o}}+\frac{\sigma_{pr}^{2}}{n_{r}}+\frac{\sigma_{po}^{2}}{n_{o}}+\frac{\sigma_{ro}^{2}}{n_{r}n_{o}}+\frac{\sigma_{e}^{2}}{n_{r}n_{o}}}$$

where $n_{r}$ is the number of raters and $n_{o}$ is the number of occasions/ratings per week. A reliability threshold of 0.70 was prespecified as acceptable. A decision (D) study was conducted via simulation by enumerating feasible combinations of $n_{r}$ × $n_{o}$ to identify minimal configurations achieving *EP*^2^ ≥ 0.70 and *EP*^2^ ≥ 0.80; when multiple solutions existed, the configuration with the smallest total number of observations ($n_{r}$ × $n_{o}$) was prioritized.

### 2.7. Delphi-Based Indicator Development: Statistical Methods

During the EPA indicator development phase, a two-round Delphi consultation was conducted. Item ratings were summarized using the mean, the SD, and the coefficient of variation (CV), and expert authority was quantified as

$$Cr=\frac{Ca+Cs}{2}$$

where *Ca* is the judgment coefficient and *Cs* is the familiarity coefficient. Inter-expert agreement was assessed using Kendall’s coefficient of concordance (Kendall’s W) with significance testing. Prespecified item retention criteria were a mean score > 3.50 and a CV < 0.25 and were supplemented by qualitative comments to refine item wording and operational descriptions. These analyses were applied to the EPA development phase only and were distinct from the longitudinal WBA validation analyses. Round-level Delphi statistics (including *Cr*, Kendall’s W, and screening decisions) are summarized in Table S3.

### 2.8. Patient and Public Involvement

Patients or the public were not involved in the design, conduct, or reporting of this research.

## 3. Results

### 3.1. Delphi-Based Indicator Development Results

A two-round Delphi consultation was completed with 21 respiratory-care experts in both rounds (response rate: 100% for each round; consultation window: November 2024 to January 2025). Expert authority remained high across rounds, with *Ca* values of 0.87 and 0.88, *Cs* values of 0.80 and 0.81, and corresponding *Cr* = (*Ca* + *Cs*)/2 values of 0.841 and 0.843 in Rounds 1 and 2, respectively (Table S3).

Inter-expert agreement was statistically significant in both rounds and improved from Round 1 to Round 2 (Kendall’s W: 0.504 vs. 0.532; both *p* < 0.01; Table S3), and the prespecified retention criteria (mean score > 3.50 and CV < 0.25) were maintained across rounds. In Round 1, 18 item-level comments and 51 description-level comments were collected, resulting in one added item and five revised items, with additional refinements to operational descriptions (four added, 15 revised, and six deleted descriptions). Specific revisions included ① adding two items, namely “Implementation of Humanistic Care and Psychological Support for Critically Ill Patients” and “Continuous Quality Improvement”; ② revising “Assisting Physicians in Rescuing Critically Ill Patients” to “Assisting Physicians in Rescuing Respiratory Critically Ill Patients”; ③ revising “Mastering In-hospital Transport Skills for Ventilated Patients” to “Safely Performing In-hospital Transport of Respiratory Critically Ill Patients”; and ④ revising “Providing Pulmonary Rehabilitation for Chronic Respiratory Diseases” to “Developing Individualized Pulmonary Rehabilitation Prescriptions.” Round 2 yielded no further structural additions and only three wording refinements. The final respiratory nursing EPA framework comprised 13 items (Table 1).

### 3.2. Sample and Assessment Overview for Clinical Validation

For longitudinal WBA validation, 30 N1-level respiratory nurses and five raters were included. Four core EPAs (EPA1, EPA2, EPA5, and EPA11) were followed over 12 study weeks (week range: 1–12), and a total of 1392 assessment events were analyzed (Table 4).

The mean number of assessments per nurse was 46.40 (SD 0.50), corresponding to 3.87 (SD 0.34) assessments per nurse per week. At the study level, the mean number of assessments per week was 116.00 (SD 0.43) (Table 4).

### 3.3. Core EPA Performance, Learning Parameters, and Attainment Trends

Core EPA performance differed across the four validated EPAs (Table 5). Event-level attainment (score ≥ 4) was the highest for EPA2 (89.1%), followed by EPA5 (86.4%), EPA1 (81.3%), and EPA11 (72.5%), and nurse-level attainment showed a similar but not identical pattern: EPA2, 24/30 (80.0%); EPA5, 22/30 (73.3%); EPA1, 19/30 (63.3%); and EPA11, 8/30 (26.7%) (Table 5).

Assessment frequency also varied by EPA. The mean number of assessments per nurse was 13.20 (SD 3.24) for EPA1, 11.60 (SD 3.86) for EPA2, 13.00 (SD 4.12) for EPA5, and 8.60 (SD 2.65) for EPA11, indicating that EPA11 had the lowest assessment frequency among the four core EPAs (Table 5).

**Figure 1:**
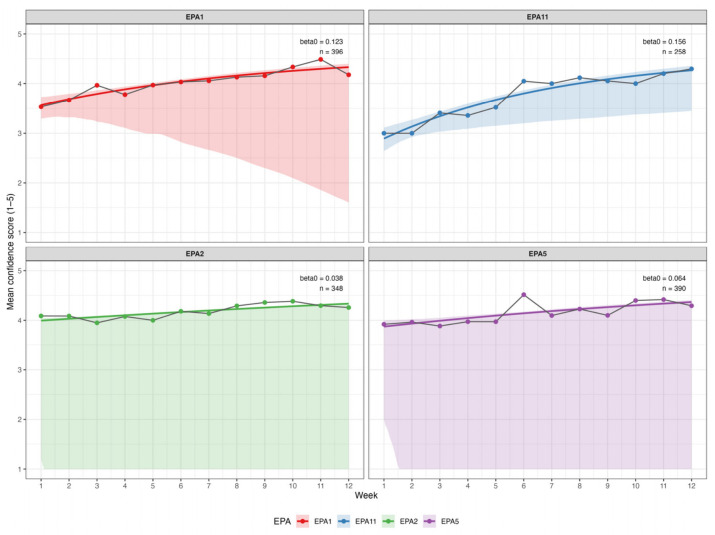
Core EPA learning curves.

Negative exponential learning-curve modeling showed distinct learning-speed profiles (Figure 1; Table 5). The estimated learning-speed parameter *β*_0_ was highest for EPA11 (0.156), followed by EPA1 (0.123), EPA5 (0.064), and EPA2 (0.038), indicating the fastest improvement trajectory for EPA11 and the slowest for EPA2. Corresponding model-based initial and asymptotic levels were 2.610 and 4.568 for EPA11, 3.430 and 4.596 for EPA1, 3.807 and 4.852 for EPA5, and 3.954 and 5.000 for EPA2 (Table 5).

**Figure 2:**
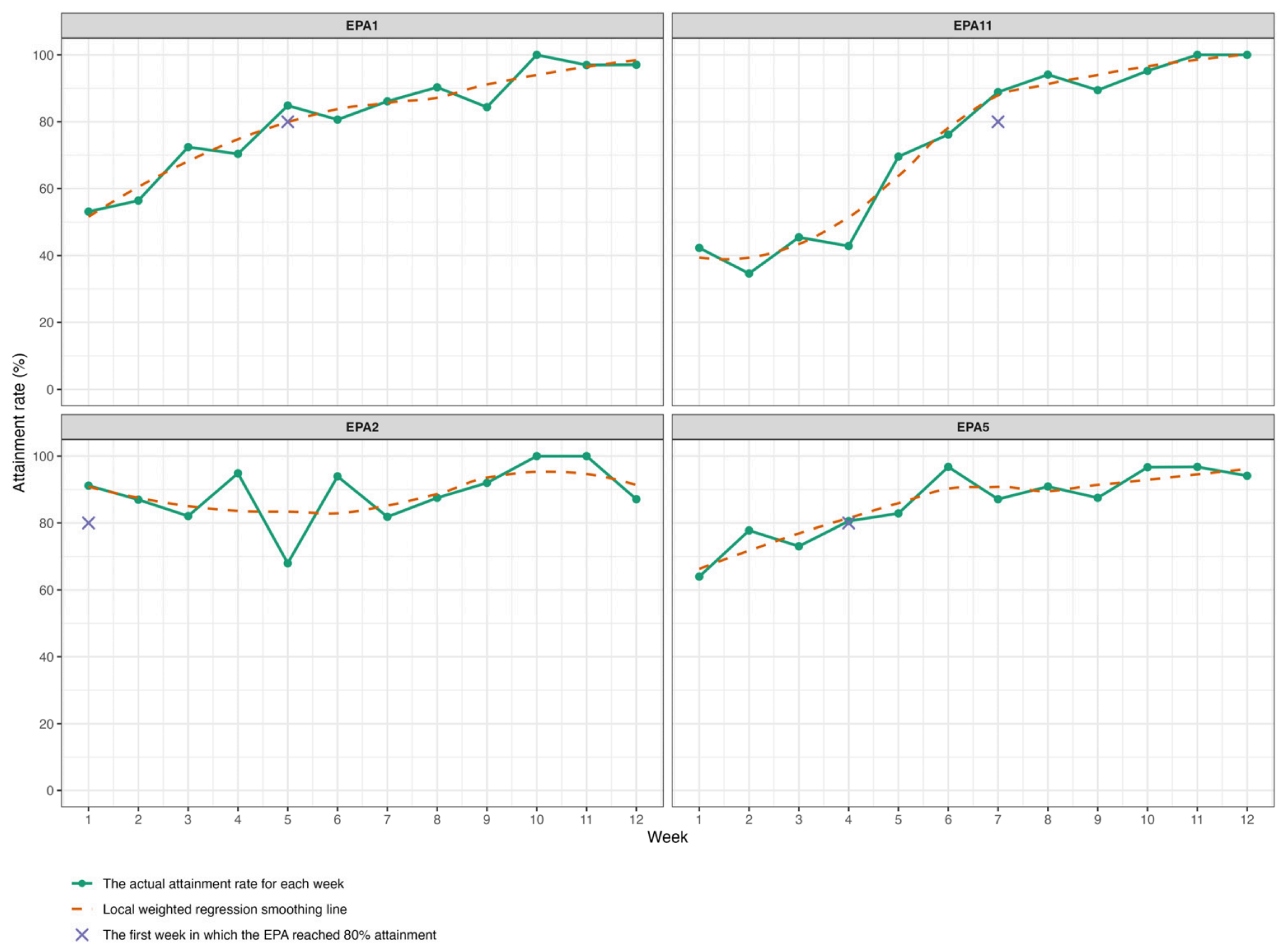
Weekly attainment rates by core EPA.

Weekly attainment trajectories are shown in Figure 2. The first observed week that reached 80% attainment was Week 1 for EPA2, Week 4 for EPA5, Week 5 for EPA1, and Week 7 for EPA11 (Figure 2; Table S4). These patterns were consistent with the lower baseline performance but faster learning speed observed for EPA11, as well as the higher baseline performance but slower slope observed for EPA2.

### 3.4. Generalizability Study and Decision Study Results

Variance decomposition in the G-study indicated that residual variance was the largest component ($\sigma_{e}^{2}$ = 0.336268, 65.05%), followed by person variance ($\sigma_{p}^{2}$ = 0.094131, 18.21%) and occasion/week variance ($\sigma_{o}^{2}$ = 0.052391, 10.13%) (Table 6). In contrast, the rater main-effect variance was small ($\sigma_{r}^{2}$ = 0.000749, 0.14%), and the person-by-rater interaction variance was similarly small ($\sigma_{pr}^{2}$ = 0.000776, 0.15%) (Table 6).

Under the observed protocol (five raters and four ratings per week), reliability coefficients met the prespecified acceptability threshold, with G = 0.792 and EP^2^ = 0.712 (Table 7). In a sensitivity protocol using five raters and 12 occasions, reliability improved further (G = 0.919, EP^2^ = 0.880; Table 7).

**Figure 3:**
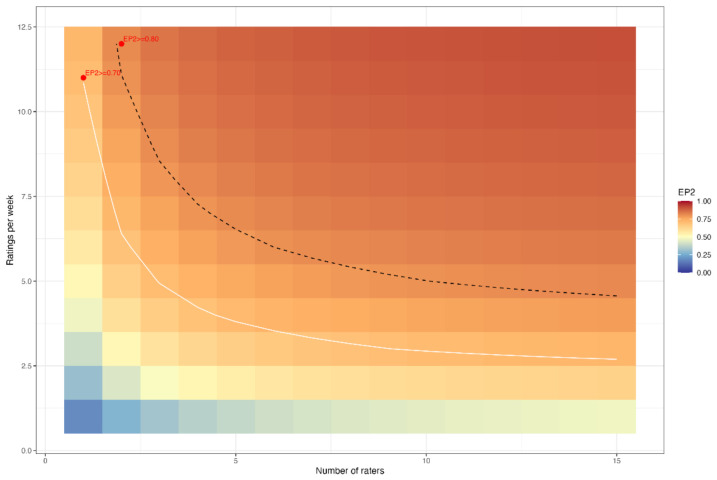
D-study decision heatmap for dependability (EP^2^).

We identified minimal feasible configurations for target dependability thresholds in the D-study (Table 8; Figure 3). For EP^2^ ≥ 0.70, the minimal configuration was one rater with 11 ratings per week (EP^2^ = 0.703; total observations = 11), while the minimal configuration was two raters with 12 ratings per week for EP^2^ ≥ 0.80 (EP^2^ = 0.812; total observations = 24). The decision heatmap (Figure 3) further illustrates the trade-off between the number of raters and ratings per week to achieve the target reliability.

### 3.5. Learning-Curve Diagnostics and Individual Heterogeneity

Model diagnostics for the negative exponential learning curves are summarized in Table S5. EPA11 showed the best overall fit (pseudo-R^2^ = 0.920) together with the fastest estimated learning speed (*β*_0_ = 0.156). EPA1 also showed a strong model fit (pseudo-R^2^ = 0.856), whereas EPA2 and EPA5 showed a more moderate fit (pseudo-R^2^ = 0.638 and 0.556, respectively) (Table S5).

Residual Shapiro–Wilk testing was non-significant for EPA1, EPA2, and EPA11 (all *p* > 0.05), whereas EPA5 showed borderline deviation from residual normality (*p* = 0.049) (Table S5). Post-peak decline flags were identified for EPA1, EPA2, and EPA5, with the first decline weeks at Weeks 12, 11, and 7, respectively; in contrast, EPA11 was not flagged for post-peak decline in the analyzed dataset (Table S5).

**Figure 4:**
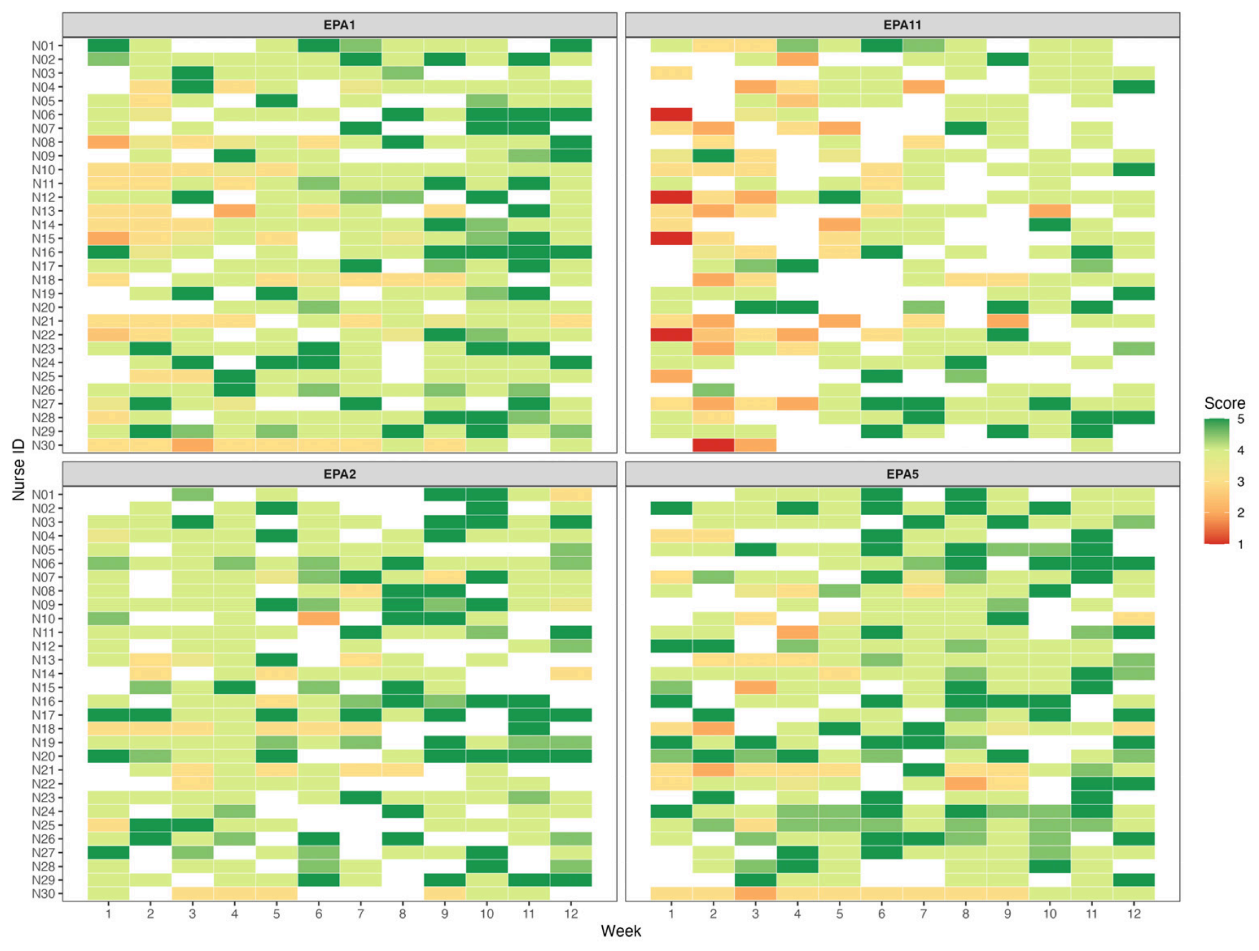
Nurse-by-week heatmaps of EPA scores.

Nurse-by-week heatmaps (Figure 4) demonstrated substantial inter-individual heterogeneity and unbalanced weekly observation density across all four EPAs while still showing an overall temporal shift toward higher entrustment scores in later study weeks. This pattern is consistent with the use of mixed-effects and G-theory approaches that accommodate unbalanced WBA data.

## 4. Discussion

In this study, we developed a respiratory nursing EPA framework through a two-round Delphi process and further conducted longitudinal clinical validation of four core EPAs using WBA data. The Delphi phase demonstrated high expert authority and significant inter-expert agreement, supporting the content validity and feasibility of the final 13-item framework. In the clinical validation phase, the four core EPAs showed distinguishable performance trajectories, acceptable dependability under the observed assessment protocol (G = 0.792, EP^2^ = 0.712), and interpretable learning-curve patterns over 12 weeks. Taken together, these findings provide multi-source evidence supporting the practical use of respiratory nursing EPAs in structured competency assessment and progression monitoring [24].

A central finding is that acceptable dependability can be achieved when EPA-aligned workplace assessments are sampled across time and assessors, even in real clinical environments with inherent variability. Recent psychometric studies implementing core EPAs and evaluating their measurement properties underscore the importance of examining score reliability and validity evidence when EPAs are used for progression decisions [21,25]. In addition, generalizability-focused work using EPA-based OSCEs demonstrates how reliability can be optimized by manipulating observation conditions, which aligns with our design-oriented interpretation of dependability [26,27].

The G-study revealed substantial residual variance (65.05%), which is not uncommon in workplace-based assessments given the inherent variability in case complexity, patient acuity, and clinical workflow [28]. Importantly, G-theory allowed us to quantify these sources rather than treating them as a single error, pointing to strategies such as enhanced rater training and structured protocols for future improvement [26]. Under the current protocol, reliability already exceeded the EP^2^ threshold (0.70), and the D-study results further showed that targets can be achieved with 1 × 11 (EP^2^ ≥ 0.70) or 2 × 12 (EP^2^ ≥ 0.80) configurations, offering flexible options for balancing staffing and dependability [27,28].

Another key finding is the heterogeneity across the four core EPAs in both attainment and learning dynamics [29]. EPA2 showed the highest event-level attainment (89.1%) and reached 80% attainment as early as Week 1, but it had the slowest learning speed (*β*_0_ = 0.038). By contrast, EPA11 had the lowest baseline and the lowest event-level attainment (72.5%) yet demonstrated the fastest estimated learning speed (*β*_0_ = 0.156) and the best model fit (pseudo-R^2^ = 0.920). This combination is educationally meaningful [22]. A lower starting point paired with a steeper trajectory may indicate that EPA11 captures a domain in which novice nurses initially have less confidence or less exposure but improve rapidly under repeated practice and feedback [30]. Conversely, a high initial attainment level with a flatter slope, as seen for EPA2, may reflect skills that are comparatively familiar at baseline, leaving less room for observable short-term gains [31].

The weekly attainment trajectories provide additional nuance beyond mean-score models. The first observed week reaching 80% attainment differed substantially across EPAs (Week 1 for EPA2, Week 4 for EPA5, Week 5 for EPA1, and Week 7 for EPA11), suggesting that different competencies stabilize at different times during early respiratory nursing practice [32]. This information may be useful for curriculum scheduling and formative feedback planning [33]. For example, competencies resembling EPA11 may benefit from intensified early supervision and targeted coaching, whereas competencies resembling EPA2 may require more attention to plateau detection, maintenance, and advanced performance refinement rather than simple threshold attainment [27].

The learning-curve diagnostics generally supported the use of negative exponential models for summarizing progression, but they also indicate that these models should be interpreted as approximations rather than as exact representations of all weekly patterns [29]. EPA11 and EPA1 showed strong model fit, whereas EPA2 and EPA5 showed a more moderate fit. In addition, EPA5 showed borderline deviation from residual normality (*p* = 0.049), and post-peak decline flags were detected for EPA1, EPA2, and EPA5. These findings likely reflect the inherent complexity of WBA trajectories, including fluctuating case complexity, uneven observation density, and short-term variability in performance opportunities [28]. Therefore, *β*_0_ is best interpreted as a summary indicator of the overall learning rate rather than a definitive statement of strictly monotonic improvement. The combination of model-based curves (Figure 1), weekly attainment trends (Figure 2), and nurse-by-week heatmaps (Figure 4) is thus a strength of the present analysis, as it captures both central tendency and individual-level heterogeneity.

The heatmaps further highlighted substantial inter-individual heterogeneity and unbalanced weekly observation density across nurse–week–EPA cells. This is expected in real-world WBA programs and supports the methodological choice of mixed-effects modeling and G-theory approaches that can accommodate unbalanced designs. Importantly, the presence of unbalanced observations does not invalidate the assessment system; rather, it underscores the need for analytic methods and programmatic decision rules that explicitly account for uneven sampling. From an implementation perspective, these visualizations can also help educators identify under-observed learners or under-assessed EPAs and adjust rotation-level assessment planning [27].

This study has several strengths. First, it integrates indicator development and clinical validation within a single framework, linking expert consensus (Delphi) with longitudinal WBA evidence [34]. Second, the analysis combines descriptive outcomes, learning-curve modeling, and G-study and D-study simulations, providing complementary evidence on feasibility, developmental sensitivity, and reliability [26]. Third, the use of auditable data-cleaning rules and reproducible R-based workflows strengthens transparency and reproducibility, which are important for future cross-site adaptation and external validation [35].

Several limitations should be acknowledged. Clinical validation was conducted in a single tertiary hospital system with 30 N1-level respiratory nurses and five raters, which may limit generalizability to other institutions, nurse levels, or specialty settings. The single-site design is particularly relevant because institution-specific factors—such as rater training culture, supervision practices, case-mix characteristics, and organizational support for workplace-based assessment—may influence both the observed reliability estimates and the learning trajectory patterns. While the 30 participants represent approximately 85% of the eligible N1-level respiratory nursing staff across both campuses of this hospital, this sample size remains modest for establishing robust psychometric generalizability. Nevertheless, this sample size is consistent with feasibility-focused pilot studies in similar educational research settings, and the primary aim of this study was to test the proof-of-concept validity of the EPA framework before scaling up to broader implementation.

Future research should prioritize multi-center validation with larger and more diverse samples across different tiers of hospitals to confirm the external validity of the EPA framework. In this regard, we are currently collaborating with multiple institutions to expand data collection; this work, however, requires substantial time and coordination and is therefore proposed as a key direction for future studies.

In conclusion, the respiratory nursing EPA framework developed in this study demonstrated strong expert-consensus support and promising longitudinal clinical validation evidence. The four core EPAs showed interpretable developmental trajectories, and the observed WBA protocol achieved acceptable dependability. These findings support the feasibility of using respiratory nursing EPAs as a structured, longitudinal approach to assessing competency and monitoring progression in clinical training settings.

## 5. Conclusions

We developed a respiratory nursing EPA framework through a structured Delphi process and provided longitudinal WBA-based clinical validation for four core EPAs. The findings showed acceptable dependability under the observed assessment protocol and demonstrated interpretable differences in attainment patterns and learning trajectories across EPAs. These results support the feasibility of implementing respiratory nursing EPAs for structured, longitudinal competency assessment and progression monitoring in clinical training.

Further multicenter studies with larger and more diverse samples are warranted to validate the full 13-item framework, refine assessment configurations, and examine generalizability across institutions and training contexts.

## Figures and Tables

**Table 1:** Respiratory care nurse confidence in professional behavior items and descriptions.

EPA Code	EPA Title	Operational Description
EPA1	Implement oxygen therapy	Use equipment correctly and manage alarms; assess CO_2_-retention risk using the ESCAPE principle; deliver individualized stepwise oxygen therapy; complete terminal disinfection.
EPA2	Perform mechanical airway-clearance techniques	Safely and effectively perform mechanical airway-clearance techniques (e.g., vibration sputum drainage, percussion, and postural drainage); follow standardized procedures; dynamically monitor effectiveness with proper documentation.
EPA3	Provide anticipatory nursing care	Conduct standardized assessments based on evidence-based guidelines; establish an early-warning system of risk; implement stratified interventions to effectively reduce complications.
EPA4	Assist physicians in resuscitating critically ill respiratory patients	Assist with intubation and defibrillation; manage device alarms and blood-gas abnormalities; call for help immediately when NEWS (National Early Warning Score) is ≥7; establish venous access and implement advanced life support with standardized medication practice.
EPA5	Implement non-invasive ventilation	Select appropriate equipment and masks; monitor vital signs and patient–ventilator synchrony; promptly manage alarms and record ventilatory parameters (mode and oxygen flow); evaluate efficacy with blood-gas and imaging findings.
EPA6	Implement invasive ventilation	Proficiently use invasive ventilators and humidification devices; monitor vital signs; perform infection prevention and control; record ventilation parameters; regularly evaluate therapeutic effect and readiness for weaning.
EPA7	Assist physicians with bronchoscopy in invasively ventilated patients	Select appropriate artificial airways and consumables; understand indications and complication management; use bronchoscopy-related medications proficiently; monitor vital signs and ventilator status; strictly follow disinfection standards and document post-procedure complications.
EPA8	Safely perform intrahospital transport of critically ill respiratory patients	Standardize critical-patient transport using strict indication assessment and full emergency-equipment preparation; monitor vital signs throughout; promptly manage emergent events; ensure seamless handover with the receiving unit.
EPA9	Provide artificial airway management	Standardize airway management by selecting suitable humidification devices; use suction catheters with a diameter < 50% of the airway diameter as needed; precisely control cuff pressure; regularly evaluate tracheostomy fixation and the stoma condition.
EPA10	Implement protocolized sedation and analgesia	Apply standardized pain assessment; optimize the analgesia and sedation strategy (minimum effective dose); follow the eCASH concept; ensure safety and comfort.
EPA11	Develop individualized pulmonary rehabilitation prescriptions	Accurately assess indications and contraindications; develop personalized plans including respiratory training, aerobic exercise, and inhaler-use guidance.
EPA12	Implementing humanistic care and psychological nursing for critically ill patients	Actively assess the psychological states of critically ill patients and their families, enhance communication, make decisions together with the patients and their families, implement reasonable nursing measures, dynamically observe changes, respect the patients and their families, and provide emotional support care.
EPA13	Continuous quality improvement	By applying management tools such as the PDCA cycle, the root cause analysis method (RCA), and the quality control circle (QCC), a patient safety quality improvement system is constructed to achieve standard process optimization and the promotion of best practices.

**Table 2:** Cross-over rater assignment for core EPA evaluations.

Evaluator	Responsible Nurses	Rotation Cycle	Evaluation Frequency
Evaluator A	Nurses 1–6	Bi-weekly rotation	4 times/week per person
Evaluator B	Nurses 7–12	Bi-weekly rotation	4 times/week per person
Evaluator C	Nurses 13–18	Bi-weekly rotation	4 times/week per person
Evaluator D	Nurses 19–24	Bi-weekly rotation	4 times/week per person
Evaluator E	Nurses 25–30	Bi-weekly rotation	4 times/week per person

**Table 3:** Three-tier evaluation schedule for core EPA assessments.

Evaluation Level	Evaluation Frequency	Evaluation Timing	Evaluation Content	Evaluation Method	Person in Charge
Department Level	4 times/week per person	Continuous	Evaluation of the 4 Core EPA Cycles	DOPS, Mini-CEX	5 Evaluators
Internal Medicine Level	Once a month	Weeks 4, 8, 12 (Wednesday)	Assessment of the 4 Core EPAs	OSCE	5 Evaluators
Nursing Department Level	Once a month	Weeks 2, 6, 10 (Friday)	Assessment of the 4 Core EPAs	OSCE	5 Evaluators

**Table 4:** Sample and assessment overview.

Variable	Value	Notes
Nurses, n	30	From the WBA dataset; no imputation
Raters, n	5	From the WBA dataset; no imputation
Core EPAs, n	4 (EPA1, EPA2, EPA5, EPA11)	Fixed core EPA set used for validation
Study weeks, range	1–12	From the WBA dataset
Total assessments (events), n	1392	Encounter-level evaluations in the final cleaned dataset
Assessments per nurse, mean (SD)	46.40 (0.50)	From the WBA dataset
Assessments per nurse per week, mean (SD)	3.87 (0.34)	From the WBA dataset
Assessments per week, mean (SD)	116.00 (0.43)	From the WBA dataset
Score scale	1–5 entrustment scale	Fixed study scoring rule
Attainment definition	score ≥ 4	Fixed study definition

Workplace-based assessment (WBA) event-level data were summarized after standardized cleaning and deduplication, and core EPAs were EPA1, EPA2, EPA5, and EPA11. Scores were recorded on a 1–5 entrustment scale, with attainment defined as a score ≥ 4. No imputation was performed.

**Table 5:** Core EPA performance and learning parameters.

Core EPA	Assessments per Nurse, Mean (SD)	Attainment Events per Nurse (Score ≥ 4), Mean (SD)	Nurse-Level Attainment, n/N (%)	Event-Level Attainment, %	Learning Speed β0	Start	Asymptote
EPA1 (n = 396)	13.20 (3.24)	10.73 (3.79)	19/30 (63.3%)	81.3	0.123	3.43	4.596
EPA2 (n = 348)	11.60 (3.86)	10.33 (4.63)	24/30 (80.0%)	89.1	0.038	3.954	5
EPA5 (n = 390)	13.00 (4.12)	11.23 (4.07)	22/30 (73.3%)	86.4	0.064	3.807	4.852
EPA11 (n = 258)	8.60 (2.65)	6.23 (2.74)	8/30 (26.7%)	72.5	0.156	2.61	4.568

Rows represent the four core EPAs. Event-level attainment was defined as a score ≥ 4, while nurse-level attainment was defined as the proportion of nurses with at least one attainment event (score ≥ 4) for the corresponding EPA. Learning trajectories were modeled using a negative exponential function; *β*_0_ denotes the learning speed (larger values indicate faster improvement), and the Start and Asym parameters are reported when available from model outputs.

**Table 6:** Variance components (G-study).

	Symbol	Variance (REML)	% of Total Variance	Interpretation
Person	$\sigma_{p}^{2}$	0.094131	18.21	Between-person variance
Rater	$\sigma_{r}^{2}$	0.000749	0.14	Rater main-effect variance
Occasion/week	$\sigma_{o}^{2}$	0.052391	10.13	Occasion/week main-effect variance
Person × Rater	$\sigma_{pr}^{2}$	0.000776	0.15	Person-by-rater interaction variance
Person × Occasion	$\sigma_{po}^{2}$	0.030971	5.99	Person-by-occasion interaction variance
Rater × Occasion	$\sigma_{ro}^{2}$	0.001688	0.33	Rater-by-occasion interaction variance
Residual	$\sigma_{e}^{2}$	0.336268	65.05	Residual/unexplained variance

Variance components were estimated using restricted maximum likelihood (REML) under a crossed-facet generalizability framework (person × rater × occasion/week), including two-way interactions and residual variance. Percentages represent the proportion of total variance attributable to each component.

**Table 7:** Reliability under specified protocols.

Protocol	*n_r_*	*n_o_*	G	EP^2^
Observed protocol (5 raters, 4 ratings/week)	5	4	0.792	0.712
Sensitivity protocol (12 occasions)	5	12	0.919	0.88

*n_r_* denotes the number of raters, while *n_o_* denotes the number of ratings/occasions per week. Generalizability (G) and dependability (EP^2^) coefficients are reported for the observed protocol and a sensitivity protocol. The prespecified threshold for acceptable reliability was 0.70.

**Table 8:** Minimal configurations for the D-study.

Target Threshold	*n_r_*	*n_o_*	EP^2^	Total Observations (*n_r_* × *n_o_*)
EP^2^ ≥ 0.70 (minimal)	1	11	0.703	11
EP^2^ ≥ 0.80 (minimal)	2	12	0.812	24

Minimal configurations to meet the target EP^2^ thresholds were selected by prioritizing the smallest total number of observations (*n_r_* × *n_o_*); if multiple solutions were tied, the configuration with the smaller *n_r_* was preferred.

## Data Availability

The datasets generated and/or analyzed during the current study are not publicly available because they contain institution-specific workplace assessment records and potentially identifiable personnel information, but they are available from the corresponding author upon reasonable request and with permission from the participating institution and ethics committee.

## Supplementary Materials

The following supporting information can be downloaded at https://www.mdpi.com/article/10.3390/healthcare14152426/s1. Table S1: Rater panel characteristics: This table summarizes rater information as reported in the source manuscript. NR indicates not reported/not specified for role-specific counts. Raters received standardized training prior to formal assessment; Table S2: EPA definitions (EPA1–EPA11): Operational definitions were translated from the original Chinese EPA indicator table and edited for concise English presentation without changing the intended meaning. Terms such as ESCAPE, NEWS, and eCASH are retained as shown in the source; Table S3: Delphi-based EPA development statistics and screening decisions: This table summarizes Delphi consultation statistics for EPA indicator development, including expert authority (*Ca*, *Cs*, and *Cr*), Kendall’s W, screening thresholds (mean > 3.50 and CV < 0.25), and revision decisions. “Not reported” indicates that item-level numerical summaries were not available as extractable source tables. Table S4: Weekly attainment by EPA: Attainment (%) was calculated as the proportion of assessments with a score ≥ 4 within each EPA–week cell. *n* denotes the number of assessments contributing to each EPA–week estimate. No imputation was performed for unobserved EPA–week combinations. Table S5: Learning model diagnostics: Per-EPA trajectories were fitted using a negative exponential model: *Score*(*t*) = *Asym* − (*Asym* − *Start*) × *exp* (−*β*_0_ × *t*). The table reports *β*_0_, pseudo-R^2^, AIC, BIC, Shapiro–Wilk *p*-values for residuals, and post-peak decline flags.

## Abbreviations

AMEE	Association for Medical Education in Europe
Ca	judgment coefficient
Cr	expert authority coefficient
Cs	familiarity coefficient
CV	coefficient of variation
D-study	decision study
EPA	entrustable professional activity
EP^2^	dependability coefficient
G-study	generalizability study
mini-CEX	Mini Clinical Evaluation Exercise
OSCE	objective structured clinical examination
REML	restricted maximum likelihood
SD	standard deviation
WBA	workplace-based assessment
