# Supplementary material for: Development and Clinical Validation of an Entrustable Professional Activities Framework for Respiratory Nurses: A Longitudinal Workplace-Based Study
Source: Healthcare (Basel). 2026 Aug 6;14(15):2426. doi: 10.3390/healthcare14152426 (PMC13463850; doi:10.3390/healthcare14152426)
Supplement: Supplementary file 1 [file healthcare-14-02426-s001.zip › healthcare-4269458-supplementary.pdf]

Supplementary material

Table S1. Rater Panel Characteristics.

This table summarizes rater information as reported in the source manuscript. NR indicates not reported/not specified for role-specific counts. Raters received standardized training prior to formal assessment.

Table S2. EPA Definitions (EPA1–EPA11).

Operational definitions were translated from the original Chinese EPA indicator table and edited for concise English presentation without changing the intended meaning. Terms such as ESCAPE, NEWS, and eCASH are retained as in the source.

Table S3. Delphi-Based EPA Development Statistics and Screening Decisions.

This table summarizes Delphi consultation statistics for EPA indicator development, including expert authority (Ca, Cs, and Cr), Kendall's W, screening thresholds (mean > 3.50 and CV < 0.25), and revision decisions. "Not reported" indicates that item-level numerical summaries were not available as extractable source tables.

Table S4. Weekly Attainment by EPA.

Attainment (%) was calculated as the proportion of assessments with score ≥ 4 within each EPA–week cell. n denotes the number of assessments contributing to each EPA–week estimate. No imputation was performed for unobserved EPA–week combinations.

Table S5. Learning Model Diagnostics.

Per-EPA trajectories were fitted using a negative exponential model: Score(t) = Asym − (Asym − Start) × exp (−β0 × t). The table reports β0, pseudo-R^2, AIC, BIC, Shapiro–Wilk p values for residuals, and post-peak decline flags.

Table S1. Rater Panel Characteristics.

This table summarizes rater information as reported in the source manuscript. NR indicates not reported/not specified for role-specific counts. Raters received standardized training prior to formal assessment.

Domain	Characteristic	Value	Remarks
**Overall team**	Total evaluators/raters	5	Reported in manuscript text
**Demographics**	Sex (female)	5 (100.0%)	n (%); all raters were female
**Demographics**	Age, years	36.0 ± 4.3	Mean ± SD; years
**Professional level**	Senior professional title	5	All raters were senior-title staff
**Role/position**	Provincial respiratory specialist nurse	NR	role-specific counts not specified
**Role/position**	Member, Respiratory Nursing Society	NR	role-specific counts not specified
**Role/position**	Head nurse, respiratory department	NR	role-specific counts not specified
**Role/position**	Chief preceptor, respiratory department	NR	role-specific counts not specified

Table S2. EPA Definitions (EPA1–EPA11).

Operational definitions were translated from the original Chinese EPA indicator table and edited for concise English presentation without changing the intended meaning. Terms such as ESCAPE, NEWS, and eCASH are retained as in the source.

EPA code	EPA title	Operational description
EPA1	Implement oxygen therapy	Use equipment correctly and manage alarms; assess CO2-retention risk using the ESCAPE principle; deliver individualized stepwise oxygen therapy; complete terminal disinfection.
EPA2	Perform mechanical airway-clearance techniques	Safely and effectively perform mechanical airway-clearance techniques (e.g., vibration sputum drainage, percussion, postural drainage); follow standardized procedures; dynamically monitor effectiveness and document completely.
EPA3	Provide anticipatory nursing care	Conduct standardized assessments based on evidence-based guidelines; establish a risk early-warning system; implement stratified interventions to effectively reduce complications.
EPA4	Assist physicians in resuscitating critically ill respiratory patients	Assist with intubation and defibrillation; manage device alarms and blood-gas abnormalities; call for help immediately when NEWS (National Early Warning Score) is >=7; establish venous access and implement advanced life support with standardized medication practice.
EPA5	Implement non-invasive ventilation	Select appropriate equipment and masks; monitor vital signs and patient-ventilator synchrony; promptly manage alarms and record ventilatory parameters (mode, oxygen flow); evaluate efficacy with blood-gas and imaging findings.
EPA6	Implement invasive ventilation	Proficiently use invasive ventilators and humidification devices; monitor vital signs; perform infection prevention and control; record ventilation parameters; regularly evaluate therapeutic effect and readiness for weaning.
EPA7	Assist physicians with bronchoscopy in invasively ventilated patients	Select appropriate artificial airway and consumables; understand indications and complication management; use bronchoscopy-related medications proficiently; monitor vital signs and ventilator status; strictly follow disinfection standards and document post-procedure complications.
EPA8	Safely perform intrahospital transport of critically ill	Standardize critical-patient transport by strict indication assessment and full emergency-equipment preparation; monitor vital signs throughout; promptly manage emergent events; ensure seamless handover with the receiving unit.

	respiratory patients	
**EPA 9**	Provide artificial airway management	Standardize airway management by selecting suitable humidification devices; use suction catheters with diameter <50% of airway diameter as needed; precisely control cuff pressure; regularly evaluate tracheostomy fixation and stoma condition.
**EPA 10**	Implement protocolized sedation and analgesia	Apply standardized pain assessment; optimize analgesia and sedation strategy (minimum effective dose); follow the eCASH concept; ensure safety and comfort.
**EPA 11**	Develop individualized pulmonary rehabilitation prescriptions	Accurately assess indications and contraindications; develop personalized plans including respiratory training, aerobic exercise, and inhaler-use guidance.

Table S3. Delphi-Based EPA Development Statistics and Screening Decisions.

This table summarizes Delphi consultation statistics for EPA indicator development, including expert authority (Ca, Cs, and Cr), Kendall's W, screening thresholds (mean > 3.50 and CV < 0.25), and revision decisions. "Not reported" indicates that item-level numerical summaries were not available as extractable source tables.

Section	Metric	Round 1	Round 2	Criterion / Formula	Notes
**A. Delphi process**	Experts (n)	21	21	-	Respiratory-care experts
**A. Delphi process**	Response rate	100%	100%	-	Effective questionnaire return rate
**A. Delphi process**	Consultation window	2024-11 to 2025-01	2024-11 to 2025-01	-	Two electronic rounds
**B. Expert authority**	Judgment coefficient (Ca)	0.87	0.88	-	Reported in manuscript
**B. Expert authority**	Familiarity coefficient (Cs)	0.8	0.81	-	Reported in manuscript
**B. Expert authority**	Authority coefficient (Cr)	0.841	0.843	Cr=(Ca+Cs)/2	High authority in both rounds
**C. Consensus**	Kendall's W	0.504	0.532	Higher W indicates stronger concordance	Agreement increased in Round 2
**C. Consensus**	P value for Kendall's W	<0.01	<0.01	Statistical significance threshold: 0.05	Both rounds statistically significant
**D. Item screening**	Retention threshold	Mean>3.50; CV<0.25	Mean>3.50; CV<0.25	Pre-specified	Applied to importance/feasibility/validity
**D. Item screening**	Item-level mean +/- SD	Not reported	Not reported	Descriptive at item/domain level	Not numerically tabulated in available source files
**D. Item screening**	Item-level CV	Reported as all <0.25	Reported as all <0.25	CV=SD/Mean	Only threshold-pass statement available

**E. Revisions and decisions**	Item-level comments	18	Not reported	-	Round-1 item modification comments
**E. Revisions and decisions**	Description-level comments	51	3 minor refinements	-	Round-2 focused on wording fine-tuning
**E. Revisions and decisions**	Structural changes	+1 item; 5 items revised	No new structural change reported	-	Based on Delphi feedback
**E. Revisions and decisions**	Description changes	+4 added; 15 revised; 6 deleted	3 wording refinements	-	Operational-description optimization
**E. Revisions and decisions**	Final EPA items	13	13	-	Finalized after Round 2

Table S4. Weekly Attainment by EPA.

Attainment (%) was calculated as the proportion of assessments with score ≥ 4 within each EPA–week cell. n denotes the number of assessments contributing to each EPA–week estimate. No imputation was performed for unobserved EPA–week combinations.

EPA	Week	n (assessments)	Attainment (%; score ≥4)
**EPA1**	1	32	53.1
**EPA1**	2	39	56.4
**EPA1**	3	29	72.4
**EPA1**	4	27	70.4
**EPA1**	5	33	84.8
**EPA1**	6	31	80.6
**EPA1**	7	36	86.1
**EPA1**	8	31	90.3
**EPA1**	9	32	84.4
**EPA1**	10	39	100
**EPA1**	11	33	97
**EPA1**	12	34	97.1
**EPA2**	1	34	91.2
**EPA2**	2	23	87
**EPA2**	3	39	82.1
**EPA2**	4	39	94.9
**EPA2**	5	25	68
**EPA2**	6	33	93.9
**EPA2**	7	22	81.8
**EPA2**	8	24	87.5
**EPA2**	9	25	92
**EPA2**	10	26	100
**EPA2**	11	27	100
**EPA2**	12	31	87.1
**EPA5**	1	25	64
**EPA5**	2	27	77.8
**EPA5**	3	26	73.1
**EPA5**	4	36	80.6
**EPA5**	5	35	82.9
**EPA5**	6	31	96.8
**EPA5**	7	31	87.1
**EPA5**	8	44	90.9
**EPA5**	9	40	87.5
**EPA5**	10	30	96.7
**EPA5**	11	31	96.8

**EPA5**	12	34	94.1
**EPA11**	1	26	42.3
**EPA11**	2	26	34.6
**EPA11**	3	22	45.5
**EPA11**	4	14	42.9
**EPA11**	5	23	69.6
**EPA11**	6	21	76.2
**EPA11**	7	27	88.9
**EPA11**	8	17	94.1
**EPA11**	9	19	89.5
**EPA11**	10	21	95.2
**EPA11**	11	25	100
**EPA11**	12	17	100

Table S5. Learning Model Diagnostics.

Per-EPA trajectories were fitted using a negative exponential model: Score(t) = Asym − (Asym − Start) × exp (−β0 × t). The table reports β0, pseudo-R², AIC, BIC, Shapiro–Wilk p values for residuals, and post-peak decline flags.

**EPA**	**n_evaluations**	**β0**	**pseudo_R2**	**AIC**	**BIC**	**residual_shapiro_p**	**decline_after_peak**	**first_decline_week**	**Interpretation**
**EPA1**	396	0.123	0.856	-13.68	-11.74	0.199	Yes	12	Decline flagged
**EPA2**	348	0.038	0.638	-18.05	-16.11	0.317	Yes	11	Decline flagged
**EPA5**	390	0.064	0.556	-5.28	-3.34	0.049	Yes	7	Decline flagged
**EPA11**	258	0.156	0.92	-7.64	-5.7	0.307	No	—	No decline
